# Prevalence and factors associated with wound colonization by *Staphylococcus spp.* and *Staphylococcus aureus* in hospitalized patients in inland northeastern Brazil: a cross-sectional study

**DOI:** 10.1186/1471-2334-14-328

**Published:** 2014-06-13

**Authors:** Gilmara Celli Maia Almeida, Marquiony Marques dos Santos, Nara Grazieli Martins Lima, Thiago André Cidral, Maria Celeste Nunes Melo, Kenio Costa Lima

**Affiliations:** 1 Department of Dentistry, University of State of Rio Grande do Norte, Caico, Brazil; 2 Microbiology Laboratory, University of State of Rio Grande do Norte, Caico, Brazil; 3 Department of Microbiology and Parasitology, Federal University of Rio Grande do Norte, Natal, Brazil; 4 Postgraduate Public Health, Department of Dentistry, Federal University of Rio Grande do Norte, Natal, Brazil

**Keywords:** *Staphylococcus spp*, *Staphylococcus aureus*, MRSA, Wounds, Hospitalization

## Abstract

**Background:**

Infections by *Staphylococcus spp.* are often associated with wounds, especially in hospitalized patients. Wounds may be the source of bacteria causing cross-contamination, and are a risk factor for methicillin-resistant *Staphylococcus aureus* (MRSA) infection. The aim of this study was to investigate the prevalence of wound colonization by *Staphylococcus spp.*, especially *S. aureus* and MRSA, in hospitalized patients, and to identify the factors associated with such colonization.

**Methods:**

This cross-sectional study enrolled patients with wounds who were hospitalized in a remote and underdeveloped inland region of northeastern Brazil with extreme poverty. Samples were collected using sterile swabs with 0.85% saline solution, and coagulase-negative *Staphylococcus spp.*, *S. aureus,* and MRSA were identified using standard laboratory procedures. Data regarding the sociodemographic characteristics, antibiotic use, and comorbidities of the patients were collected using the medical records and a questionnaire.

**Results:**

A total of 125 wounds were analyzed. The patients had a mean age of 63.88 years and a mean 3.84 years of school education. Eighty-one wounds (64.80%) were colonized by *Staphylococcus spp.* Twenty-five wounds (20%) were colonized by *S. aureus*, 32% of which were colonized by MRSA. Wound colonization by *Staphylococcus spp.* was associated with pneumonia or other respiratory disease (p = 0.03). Wound colonization by *S. aureus* was associated with nasal colonization by *S. aureus* (p < 0.001), fewer days of prior antibiotic use (p = 0.04), admission to a medical ward (p = 0.02), and age >65 years (p = 0.05). Among patients with wound colonization by MRSA, 37.50% had a history of prior antibiotic use, 75% had two or more comorbidities, 25% had cancer or diabetes, 50% had cardiovascular disease, and 50% died.

**Conclusions:**

Wounds can be the source of *Staphylococcus spp.* infection, and high proportions of wounds are colonized by *S. aureus* and MRSA. Nasal colonization by *S. aureus* may be a source for wound colonization by *S. aureus*, illustrating the importance of preventing cross-contamination in hospital environments, especially among elderly patients. Wounds should be carefully managed to prevent microbial spread, thereby assisting patient recovery and reducing healthcare costs.

## Background

Infections by *Staphylococcus spp.* are common in developing countries [1]. Variations in antimicrobial susceptibility make it difficult to determine the infection patterns of *Staphylococcus spp.* in socioeconomically underdeveloped regions [2,3]. Hospitalization of patients increases their risk of infection, and hospital-acquired infections are associated with higher mortality rates, longer hospitalization periods, and increased healthcare costs compared with community-acquired infections [4,5].

The wounds of hospitalized patients may result from surgery, pressure ulcers, diabetic ulcers, or hospital- or community-acquired injuries, and wound infections can result in recurrent hospitalization. The relative frequencies of organisms causing wound infections varying greatly among studies. *Staphylococcus aureus* and *Pseudomonas aeruginosa* are among the organisms most commonly isolated from severe wounds, and colonization by these organisms requires careful management because of their ability to acquire antibiotic resistance and their association with nosocomial infections [6-13]. Wounds are a risk factor for colonization with methicillin-resistant *S. aureus* (MRSA) and other multidrug-resistant organisms, especially in hospital environments [14-18]. Bacterial colonization of wounds can increase wound severity and interfere with healing [6,11].

Even though skin and soft tissue infections are common in hospitalized patients [19], few epidemiological or clinical studies of these infections have been reported [4], especially studies focusing on the characteristics of specific healthcare systems or regions. Infections with antibiotic-resistant bacteria are associated with prolonged hospitalization, increased morbidity and mortality, and increased healthcare costs [4,5,20]. Investigation of the pattern of infection by *Staphylococcus spp.* in a hospital environment is therefore warranted, especially in wounds, which may be a source of cross-contamination by MRSA and coagulase-negative *Staphylococcus spp.*[11,15,18].

Lack of infrastructure and research funding have resulted in a lack of healthcare studies in northeastern Brazil, which has some of the poorest socioeconomic conditions in the country [21], especially in inland regions that are far from state capitals. The aim of the present study was to investigate the prevalence of wound colonization by *Staphylococcus sp.*, especially *S. aureus* and MRSA, in patients admitted to a public hospital in an inland region of northeastern Brazil, and to evaluate the factors associated with such colonization.

## Methods

### Study area and design

This 1-year cross-sectional study enrolled patients with wounds who were admitted to the Hospital Regional do Serido, municipality of Caico, state of Rio Grande do Norte, Brazil. Caico is located in an inland region of northeastern Brazil and covers an area of 1228.6 km^2^. The Hospital Regional do Serido is a referral hospital for patients from the inland part of Rio Grande do Norte, which treats patients from more than 14 municipalities, with an average of 2,000 admissions per year. The hospital includes 29 medical beds, 47 surgical beds, and 5 intensive care unit (ICU) beds. The study population lives in a remote and underdeveloped region.

### Study population, sample size, and sampling procedures

All patients aged 18 years or older who were admitted to the medical ward, surgical ward, or ICU of the Hospital Regional do Serido with one or more infected wounds on days allocated for data gathering during 2012 were included in the study. Patients with wounds treated only in the outpatient clinics were not included in the study. Most patients (71.80%) had only one wound, and the remaining patients had two or more wounds each. As each wound was swabbed and included in the study, some patients were included more than once. The sampling unit of the survey was the wound. Data collection occurred on Monday, Tuesday, and Wednesday of each week, to enable performance of routine laboratory procedures. A total of 125 infected wounds were swabbed. Nasal swabs were also taken from all enrolled patients, as nasal colonization by *Staphylococcus spp.* and *S. aureus* were independent variables in the study.

### Data collection procedures and collection of microbiological samples

A structured questionnaire was used to collect socioeconomic and demographic data from patients, including age, sex, years of school education, area of residence (rural or urban), marital status, number of children, and number of consumer goods available at home (car or motorcycle, internet access, and electronic goods including television, mobile phone, computer, DVD player, and stereo). The questionnaire also collected data regarding prior hospital admissions (within the past year), prior antibiotic use (within the past 6 months), and the time the wound was acquired. The medical records were reviewed to obtain information regarding the time of hospitalization, reason for hospitalization, antibiotic use, wound characteristics, and comorbidities. Chronic diseases (such as diabetes mellitus, hypertension, and liver dysfunction), conditions causing immunocompromise (such as organ transplantation, immunosuppressant medication, cancer, HIV infection, leukemia, and anemia), and opportunistic diseases were recorded.

After debridement of the wound edges and cleaning with physiological saline, the deepest part of the wound was swabbed with a sterile swab dipped in 0.85% saline solution. Both nostrils of each patient were also swabbed with a sterile swab dipped in 0.85% saline solution. The diameter and characteristics (pus, edema, erythema, and necrosis) of each wound, the hospitalization period, and the clinical outcome (discharge, transfer, or death) were recorded.

### Culture and microbial identification

All swabs were placed in sterile tubes containing brain heart infusion broth, and transported to the microbiology laboratory of the Caico Campus of the Universidade do Estado do Rio Grande do Norte in a cooler with ice. At the laboratory, the samples were incubated at 37°C for 24 h, after which 0.1-mL samples were seeded onto mannitol salt agar and incubated in a bacterial incubator at 37°C for 48 h. Colonies suggestive of staphylococci underwent Gram staining and catalase and coagulase testing. Colonies with Gram-positive cocci that were catalase-positive and coagulase-positive were classified as presumptive *S. aureus*, and colonies with Gram-positive cocci that were coagulase-negative were classified as coagulase-negative *Staphylococcus spp.*[22]. As it was not possible to perform polymerase chain reaction (PCR) amplification to confirm the species at this stage, colonies were classified as presumptive *S. aureus*, and PCR was performed for MRSA strains only.

### MRSA screening and PCR amplification for detection of the *mecA* gene

Colonies identified as presumptive *S. aureus* underwent susceptibility testing using the disc diffusion method in Mueller–Hinton agar. Resistance to cefoxitin 30 g (DME-Sensidisc®, Brazil) was tested to identify colonies as MRSA or methicillin-sensitive *S. aureus*, in accordance with the Clinical and Laboratory Standards Institute guidelines (2013) [22]. *S. aureus* ATCC 25923 was used as the quality control for antimicrobial susceptibility tests. To identify MRSA colonies, total DNA extraction was performed on the samples that were resistant to cefoxitin [23], followed by PCR amplification for detection of the *mecA* gene as described by Oliveira and De Lencastre in 2002 [24].

### Statistical analysis

Wound colonization with *Staphylococcus spp.* and *S. aureus* were considered as dependent variables. Relationships between variables were analyzed using the chi-square test or Fisher's exact test, with the crude prevalence ratio used to determine the magnitude of associations. To analyze the relationships between variables, the quantitative variables were converted to categorical variables using their median values. Multivariable analysis was performed using the Poisson regression model with robust variance [25,26], including the variables with values of p < 0.20 on bivariate analysis and important adjustment variables. The adjusted prevalence ratio was used to determine the magnitude of associations on multivariable analysis. Differences were considered significant at p < 0.05 in all tests. All statistical analyses were performed using Stata statistical software, release 10.0 (College Station, TX).

### Ethical considerations

All patients gave written informed consent for inclusion in the study. The study was approved by the Research Ethics Committee of the Universidade do Estado do Rio Grande do Norte (protocol number 001/11).

## Results

The sociodemographic characteristics of the enrolled patients are shown in Table 1. There were no significant associations between sociodemographic characteristics and wound colonization by *Staphylococcus spp.* or *S. aureus*. Eighty-five of the 125 wounds swabbed (68%) were from patients in the medical ward, 20 (16%) were from patients in the surgical ward, and 20 (16%) were from patients in the ICU. The mean period of hospitalization was 8.60 ± 6.35 days, and the mean number of antibiotics used was 2.08 ± 0.92. Among patients with wound colonization by *Staphylococcus spp*., 72% were prescribed more than one antibiotic, including a cephalosporin in 90% of cases (usually third or fourth generation), ciprofloxacin in 48% of cases, and oxacillin in 23% of cases. However, there was no established pattern for the treatment of wound colonization by *Staphylococcus spp*., with more than 20 antibiotics in eight groups used.

**Table 1:** Sociodemographic characteristics of hospitalized patients

**Sociodemographic characteristics**	**n (%) or Mean ± SD**
**Sex**	
Male	63 (50.40)
Female	62 (49.60)
**Marital status**	
Married	47 (37.60)
Single/separated/divorced	40 (32.00)
Widowed	38 (30.40)
**Area of residence**	
Rural	20 (16.00)
Urban	105 (84.00)
**Age**	63.88 ± 18.57
**School education (years)**	3.84 ± 3.70
**Persons per household**	4.40 ± 3.12
**Children per household**	3.18 ± 3.23
**Number of consumer goods**	8.00 ± 3.95

Urban area = inside the city limits of a town or village, defined by a municipal area. Rural area = a municipality outside the city limits. An area of agricultural activities and livestock, rural tourism, forestry, or environmental conservation (Instituto Brasileiro de Geografia e Estatística).

Among wounds colonized by *Staphylococcus spp*., 79 (63.20%) were in patients with prior hospitalization, 49 (39.20%) were in patients with prior antibiotic use, 59 (47.20%) were in patients with diabetes mellitus, 66 (52.80%) were in patients with cardiovascular disease, 26 (20.80%) were in patients with pneumonia or other respiratory disease, 18 (14.40%) were in patients with cancer, 35 (28%) were in patients with paralysis or other mobility disorder, and 18 (14.40%) were in patients with septicemia. The mean wound diameter was 7 ± 6.19 cm, and 63 wounds (50.40%) had been acquired within the past 30 days. Only 21 (25.92%) of the wounds colonized by *Staphylococcus spp*. were acquired in hospital.

The prevalence of *Staphylococcus spp.* colonization in wounds was 64.80% (n = 81; 95% confidence interval [CI] 56.40–73.20), the prevalence of *S. aureus* colonization was 20% (n = 25; 95% CI 12.98–27.02), and the prevalence of MRSA colonization was 6.40% (n = 8; 95% CI 6.30–6.50). Among wounds colonized by *Staphylococcus spp.*, 30.86% were colonized by *S. aureus*, of which 32% were colonized by MRSA. The majority (n = 5; 62.50%) of wounds colonized by MRSA were in female patients, and 2 (25%) were in patients with prior hospitalization. Among wounds colonized by MRSA, 3 (37.50%) were in patients with prior antibiotic use, 6 (75%) were in patients with two or more comorbidities, 2 (25%) were in patients with cancer or diabetes mellitus, 4 (50%) were in patients with cardiovascular disease, 6 (75%) were in patients who received two or more antibiotics, and 4 (50%) were in patients who died. Among wounds colonized by MRSA, necrosis occurred in 4 (50%) and septicemia occurred in 1 (12.50%). All patients with wound colonization by MRSA also had nasal colonization by MRSA. Most of the wounds (n = 6; 75%) colonized by MRSA were acquired in the community.

There were no significant associations between wound colonization by *Staphylococcus spp*. and comorbidities, except for pneumonia or other respiratory disease (p = 0.03). There were also no significant associations between wound colonization by *S. aureus* and comorbidities. However, there were significant associations between nasal colonization by *S. aureus* and wound colonization by *Staphylococcus spp.* (p = 0.05) and *S. aureus* (p = 0.003). The relationships between wound colonization by *Staphylococcus spp.* and *S. aureus* and hospitalization variables and wound characteristics are shown Table 2. Wound colonization by *S. aureus* was significantly associated with nasal colonization by *S. aureus*, fewer days of prior antibiotic use, and admission to the medical ward. Acquisition of the wound in the community was close to the threshold for significant association with wound colonization by *S. aureus*.

**Table 2:** **Associations between wound colonization by *Staphylococcus spp. *** **and *Staphylococcus aureus, *** **and hospitalization variables and wound characteristics**

	**Wound colonization by *Staphylococcus spp*.**				**Wound colonization by *S. aureus***			
	**Yes**	**No**	**p**	**PR**	**Yes**	**No**	**p**	**PR**
	**n (%)**	**n (%)**		**(95% CI)**	**n (%)**	**n (%)**		**(95% CI)**
**Type of ward**								
Medical	55 (65.90)	29 (34.10)	0.87	1.05	22 (25.90)	63 (74.10)	0.03	3.45
Surgical or ICU	25 (62.50)	15 (37.50)		(0.79-1.40)	3 (7.50)	37 (92.50)		(1.10-10.86)
**Length of hospitalization**								
≥7 days	36 (57.10)	27 (42.90)	0.10	0.79	10 (15.90)	53 (84.10)	0.35	0.66
<7 days	45 (72.60)	17 (27.40)		(0.60-1.02)	15 (24.20)	47 (75.80)		(0.32-1.35)
**Prior antibiotic use**								
≥3 days	37 (58.70)	26 (41.30)	0.21	0.83	6 (9.50)	57 (90.50)	0.01	0.31
<3 days	44 (71.00)	18 (29.00)		(0.64-1.07)	19 (30.60)	43 (69.40)		(0.13-0.73)
**Clinical outcome**								
Death	31 (64.60)	17 (35.40)	1.00	1.01	11 (22.90)	37 (77.10)	0.60	1.32
Discharge or transfer	48 (64.00)	27 (36.00)		(0.77-1.32)	13 (17.30)	62 (82.70)		(0.65-2.71)
**Prior hospitalization**								
Yes	49 (62.00)	30 (38.00)	0.51	0.89	12 (15.20)	67 (84.80)	0.13	0.54
No	32 (69.60)	14 (30.40)		(0.69-1.15)	13 (28.30)	33 (11.70)		(0.27-1.08)
**Prior antibiotic use**								
Yes	28 (57.10)	21 (42.90)	0.21	0.82	8 (16.30)	41 (83.70)	0.55	0.73
No	53 (69.70)	23 (30.30)		(0.62-1.09)	17 (22.40)	59 (77.60)		(0.34-1.56)
**Wound acquisition**								
Hospital	21 (67.70)	10 (32.30)	0.86	1.06	2 (6.50)	29 (93.50)	0.05	0.26
Community	60 (63.80)	34 (36.20)		(0.80-1.41)	23 (24.50)	71 (75.50)		(0.07-1.05)
**Wound type**								
Pressure ulcer	40 (63.50)	23 (36.50)	0.90	0.96	11 (17.50)	52 (82.50)	0.62	0.77
Diabetic foot or other	41 (66.10)	21 (33.90)		(0.74-1.24)	14 (22.60)	48 (77.40)		(0.38-1.57)
**Wound site**								
Lower limb	43 (66.20)	22 (33.80)	0.89	1.04	17 (26.20)	48 (73.80)	0.12	1.96
Sacral or other	38 (63.30)	22 (36.70)		(0.81-1.35)	8 (13.30)	52 (86.70)		(0.91-4.21)
**Wound duration**								
≤days	43 (68.30)	20 (31.70)	0.53	1.11	10 (15.90)	53 (84.10)	0.35	0.66
>30 days	38 (61.30)	24 (38.70)		(0.86-1.44)	15 (24.20)	47 (75.80)		(0.32-1.35)
**Wound diameter**								
>5 cm	36 (58.10)	26 (41.90)	0.17	0.81	13 (21.00)	49 (79.00)	0.79	1.10
≤5 cm	45 (71.40)	18 (28.60)		(0.62-1.06)	12 (19.00)	51 (81.00)		(0.55-2.22)
**Wound pus**								
Yes	22 (71.00)	9 (29.00)	0.54	1.13	9 (29.00)	22 (71.00)	0.23	1.71
No	59 (62.80)	35 (37.20)		(0.86-1.49)	16 (17.00)	78 (83.00)		(0.80-3.46)
**Wound necrosis**								
Yes	41 (57.70)	30 (42.30)	0.09	0.78	13 (18.30)	58 (81.70)	0.75	0.82
No	40 (74.10)	14 (25.90)		(0.60-1.00)	12 (22.20)	42 (77.80)		(0.41-1.66)
**Foul wound odor**								
Yes	13 (56.50)	10 (43.50)	0.50	0.85	6 (26.10)	17 (73.90)	0.40	1.40
No	68 (66.70)	34 (33.30)		(0.58-1.24)	19 (18.60)	83 (81.40)		(0.63-3.11)

Although there were no significantly associations between wound colonization by *Staphylococcus spp.* or *S. aureus* and the duration of hospitalization, the hospitalization period was longer in patients with hospital-acquired wounds than in patients with community-acquired wounds (p = 0.04).

The multivariable analysis included sex in addition to the variables with p < 0.20 on bivariate analysis, as this was an important adjustment variable in multiple models. Multivariable analysis found that wound colonization by *S. aureus* was independently associated with nasal colonization by *S. aureus*, fewer days of prior antibiotic use, and admission to the medical ward. Age was close to the threshold for independent association with wound colonization by *S. aureus* (Table 3).

**Table 3:** **Multivariable analysis of factors associated with wound colonization by *Staphylococcus aureus***

**Independent variables**		**Wound colonization by *S. aureus***		**p**	**PR (95% CI)**	**p**	**PR**_**adjusted **_**(95% CI)**
		**Absent**	**Present**				
		**n (%)**	**n (%)**				
**Nasal *S. aureus***	Present	6 (28.60)	15 (71.40)	<0.001	7.43	<0.001	4.39
	Absent	94 (90.40)	10 (9.60)		(3.88-14.21)		(2.22-8.66)
**Prior antibiotic use**	≥3 days	57 (90.50)	6 (9.50)	0.01	0.31	0.04	0.49
	<33 days	43 (69.40)	19 (30.60)		(0.13-0.73)		(0.25-0.99)
**Type of ward**	Medical	63 (74.10)	22 (25.90)	0.03	3.45	0.02	3.06
	Surgical or ICU	37 (92.50)	3 (7.50)		(1.10-10.86)		(1.15-8.14)
**Marital status**	Unmarried	58 (74.40)	20 (25.60)	0.07	2.41	0.64	1.27
	Married	42 (89.40)	5 (10.60)		(0.97-5.99)		(0.46-3.45)
**Wound acquisition**	Hospital	29 (93.50)	2 (6.50)	0.05	0.26	0.16	0.38
	Community	71 (75.50)	23 (24.50)		(0.07-1.05)		(0.10-1.46)
**Prior hospitalization**	Yes	67 (84.80)	12 (15.20)	0.13	0.54	0.40	0.76
	No	33 (71.70)	13 (28.30)		(0.27-1.08)		(0.40-1.44)
**Age**	≥65 years	46 (74.20)	16 (25.80)	0.17	1.81	0.05	1.92
	<65 years	54 (85.70)	9 (14.30)		(0.86-3.78)		(0.99-3.75)
**Sex**	Male	48 (76.20)	15 (23.80)	0.39	1.48	0.90	1.04
	Female	52 (83.90)	10 (16.10)		(0.72-3.30)		(0.52-2.10)

Model fit (chi-square test): p = 0.79.

## Discussion

The frequency of wound colonization by *S. aureus* was lower in the present study than the frequency of >30% in the majority of previously reported studies [4,9,11,27,28]. This can be attributed to almost 40% of the wounds in the present study being in patients with prior antibiotic use, of which more than 60% had a history of prior hospitalization, which may have decreased the rate of colonization by methicillin-sensitive *S. aureus*. However, slightly over 30% of the wounds colonized by *Staphylococcus spp.* were colonized by *S. aureus*, which is higher than the proportion of 18% reported in a study of isolates from blood cultures and secretions of hospitalized patients in a teaching hospital in Natal, the capital city of the state where the present study was conducted [29]. This confirms that regional characteristics of the population and healthcare processes influence the microbial profiles of hospitals.

In this study, slightly over 30% of the wounds colonized by *S. aureus* were colonized by MRSA, which is similar to previously reported findings [3,28,30-32]. Even though the present study found a lower rate of wound colonization by *S. aureus* than in studies conducted in other regions and other countries, the rate of antibiotic resistance was high, which may be attributed to prior antibiotic use by more than a third of the patients, the majority of whom were elderly with comorbidities, and use of multiple antibiotics. Some of the patients also had a history of prior hospitalization. All of these factors may have resulted in selection of weaker bacterial strains, which are generally associated with the presence of MRSA [33-35]. The colonization of wounds by MRSA indicates a need for greater control to ensure the proper use of antibiotics, and careful management of hospitalized patients and the hospital environment to avoid cross-contamination and the spread of antibiotic-resistant bacteria in the hospital. In the present study, half of the wounds colonized by MRSA were in patients with cardiac disease, and the great majority were in patients with two or more comorbidities.

It is worth noting that *Staphylococcus spp*. are not the only organisms that colonize wounds. Wound infections can also be caused by various other microorganisms, such as group A streptococci (particularly *Streptococcus pyogenes*), *Escherichia coli*, *Pseudomonas aeruginosa,* and other Gram-negative bacteria, which may present in isolation or may cause polymicrobial infections that include *Staphylococcus spp*. [36-39].

A study conducted in Denmark found that patients with nasal colonization by *S. aureus* had the same clone of *S. aureus* in their chronic ulcers [40], indicating that nasal colonization is an important source of wound contamination in the same patient, as well as cross-contamination among patients. In the present study, nasal colonization by *S. aureus* was associated with wound colonization by *Staphylococcus spp* and *S. aureus*, and all patients with wound colonization by MRSA also had nasal colonization by MRSA. These results indicate that nasal colonization is an important source of cross-contamination by multidrug-resistant bacterial strains [41].

Some studies reported that community-acquired wounds are often poorly managed and may be colonized by multidrug-resistant organisms [42,43]. Most of the wounds included in the present study were acquired in the community, which may contribute to the high frequency of colonization by antibiotic-resistant strains. However, it is difficult to determine whether colonization by *Staphylococcus spp*. occurred in the community or the hospital, even when the wounds were acquired in the community, because most patients had a history of prior hospitalization and the great majority had other systemic diseases. To determine that colonization by MRSA is community-acquired, MRSA must be isolated from a patient who has not recently been hospitalized, used antibiotics, or had a catheter placed [44].

In the present study, half of the wounds colonized by MRSA were in patients who died, and the majority were in patients with two or more comorbidities, and who received two or more antibiotics during their hospitalization. This illustrates the relationship between colonization by antibiotic-resistant bacterial strains and the presence of comorbidities that cause systemic vulnerability. Educational campaigns can reduce the spread of MRSA in the community [43], and such campaigns should be promoted in public hospitals, especially when the population has a limited education. The patients enrolled in the present study had a mean period of education of 4 years, and more than a third of the local population works in agriculture.

Strategies for prevention and control of infection are very important in the region studied, where wound infections caused by MRSA are not common and many patients with wound colonization by MRSA have previously been hospitalized or used antibiotics. Moreover, patients with wound colonization by MRSA had a high mortality rate. It is therefore necessary to investigate the routines of the staff responsible for wound care, and use the results of such investigations to promote training regarding the prevention of cross-contamination in hospitals.

The present study provides a profile of wound colonization by *Staphylococcus spp*. in patients in an undeveloped region of Brazil with extreme poverty. However, the study has some important limitations. PCR amplification for identification of *S. aureus,* and molecular type classification for identification of MRSA strains were not performed. The nasal swab results may give an indication of the prevalence of methicillin-sensitive *S. aureus* and MRSA in the community, but the MRSA strains in the community were not identified. Other skin areas could be also have been investigated as potential carriers of MRSA. Moreover, it was not possible to collect swabs on all days of the week, or to include wounds treated in outpatient clinics, which may have introduced selection bias.

## Conclusions

The present study found a high frequency of wound colonization by *Staphylococcus spp.* and moderate frequency of wound colonization by *S. aureus*. The proportion of wounds colonized by MRSA was high in this study compared with other studies. Knowledge about the colonization patterns of these microorganisms in the hospital environment is important, because of their ability to acquire resistance, which can lead to prolonged hospitalization and treatment difficulty. Nasal colonization by *S. aureus* is an important source of wound colonization by *S. aureus*, which illustrates the importance of preventing cross-contamination. Our results reinforce the need for prevention and control of hospital- and community-acquired infections, especially among elderly patients, who may need longer treatment and larger numbers of medications, and may have more comorbidities, than younger patients. In conclusion, wounds are a source of *Staphylococcus spp.* infection, and should be the focus of prevention and control strategies to avoid microbial spread, assist in patient treatment, and minimize hospitalization costs.

## Competing interests

The authors declare that they have no competing interests.

## Authors’ contributions

GCMA participated in all stages of the research (design, data collection, data analysis, and interpretation of data) and drafted the manuscript. MMS, NGML, and TAC assisted with data collection and participated in the laboratory analyses. MCNM and KCL helped with the conception and design of the study and the analysis and interpretation of data, participated in critical revision of the manuscript, and contributed to the final version of the manuscript. All authors read and approved the final manuscript.

## Pre-publication history

The pre-publication history for this paper can be accessed here:

http://www.biomedcentral.com/1471-2334/14/328/prepub
